# Copper Nanoparticles Confined in a Silica Nanochannel Film for the Electrochemical Detection of Nitrate Ions in Water Samples

**DOI:** 10.3390/molecules28227515

**Published:** 2023-11-10

**Authors:** Dewang Li, Shuai Xu, Haiyan Jin, Jinqing Wang, Fei Yan

**Affiliations:** 1 Donghai Laboratory, Zhoushan 316021, China; dwli@sio.org.cn (D.L.); jinhaiyan@sio.org.cn (H.J.); 2 Key Laboratory of Marine Ecosystem Dynamics, Second Institute of Oceanography, Ministry of Natural Resources, Hangzhou 310012, China; 3 School of Chemistry and Chemical Engineering, Zhejiang Sci-Tech University, Hangzhou 310018, China; 202120104178@mails.zstu.edu.cn; 4 College of Metrology and Measurement Engineering, China Jiliang University, Hangzhou 310018, China; jqwang@cjlu.edu.cn

**Keywords:** copper nanoparticles, silica nanochannel film, nitrate ions, electrochemical sensor, water samples

## Abstract

The nitrate ion (NO_3_^−^) is a typical pollutant in environmental samples, posing a threat to the aquatic ecosystem and human health. Therefore, rapid and accurate detection of NO_3_^−^ is crucial for both the aquatic sciences and government regulations. Here we report the fabrication of an amino-functionalized, vertically ordered mesoporous silica film (NH_2_-VMSF) confining localized copper nanoparticles (CuNPs) for the electrochemical detection of NO_3_^−^. NH_2_-VMSF-carrying amino groups possess an ordered perpendicular nanochannel structure and ultrasmall nanopores, enabling the confined growth of CuNPs through the electrodeposition method. The resulting CuNPs/NH_2_-VMSF-modified indium tin oxide (ITO) electrode (CuNPs/NH_2_-VMSF/ITO) combines the electrocatalytic reduction ability of CuNPs and the electrostatic attraction capacity of NH_2_-VMSF towards NO_3_^−^. Thus, it is a rapid and sensitive electrochemical method for the determination of NO_3_^−^ with a wide linear detection range of 5.0–1000 μM and a low detection limit of 2.3 μM. Direct electrochemical detection of NO_3_^−^ in water samples (tap water, lake water, seawater, and rainwater) with acceptable recoveries ranging from 97.8% to 109% was performed, demonstrating that the proposed CuNPs/NH_2_-VMSF/ITO sensor has excellent reproducibility, regeneration, and anti-interference abilities.

## 1. Introduction

In the past few decades, nitrate ion (NO_3_^−^) pollution in water environments has become a serious global environmental issue [1]. The wide-spread use of nitrogen fertilizers and fossil fuels dramatically increases food production and the human population. However, the excess nitrogen on land and in the air has adverse effects on aquatic ecosystems and human health. Excessive use of nitrogen-containing fertilizers in agriculture and livestock farming, along with the uncontrolled discharge of wastewater into the groundwater, can result in the contamination of multiple aquatic environments [2]. Emissions of industrial gases are also a source of nitrate pollution, which can form nitric acid and further acidify lakes and streams through atmospheric deposition [3]. Increased nitrogen flowing into estuarine and nearshore waters contributes to the eutrophication of coastal waters. This phenomenon leads to a rise in the occurrence of harmful algal blooms, coastal hypoxia, and the degradation of habitats [4]. The ultimate oxidation product of inorganic nitrogen, NO_3_^−^, accounts for more than 65% of the dissolved nitrogen in seawater. Its status and fate are crucial to the marine biogeochemical cycles of carbon [5], whose coupling regulates the climate on Earth [6]. As for human health, high levels of NO_3_^−^ intake in the human body induce methemoglobinemia, colorectal cancer, thyroid disease, and neural tube defects [7]. For these reasons, the World Health Organization, the Food and Agriculture Organization, and governments have all set strict limits on NO_3_^−^ in drinking water [8,9]. Therefore, simple and sensitive methods for the quantitative analysis of NO_3_^−^ in aquatic environments are urgently needed for the aquatic science community and governments.

Various methods for NO_3_^−^ determination include the visible spectrophotometric method using color reagents [10], direct ultraviolet spectrophotometry [11], fluorescence [12], chemiluminescence [13], electrochemical sensors [14], high-performance liquid chromatography [15], and ion chromatography [16]. Electrochemical sensing has gained widespread popularity because it generally offers a fast response time, high sensitivity, space-saving designs, and cost-efficiency [17,18,19]. However, electrochemical process of NO_3_^−^ at the common electrodes is rather slow and produces high overpotentials. To overcome this issue, researchers have focused on the exploitation of various nanomaterials, such as metal nanoparticles [20], carbon nanotubes/fibers [21], and graphene-based materials [22]. Copper nanoparticles (CuNPs) have been reported as an effective electrocatalytic material for NO_3_^−^ and nitrite (NO_2_^−^) reduction and have been combined with other carbonaceous materials for the sensitive determination of NO_3_^−^ and NO_2_^−^ [23,24,25].

Recently, mesoporous materials integrated with various functional nanomaterials have shown great potential in the fields of adsorption, catalysis, and sensing [26,27,28,29,30,31]. Vertically ordered mesoporous silica film (VMSF, also referred to as silica nanochannel film) has opened a vast range of potential opportunities for the electrochemical analysis of complicated real samples in recent decades [32,33,34]. VMSFs consisting of vertically oriented, open nanochannels attached to the electrode ensure the accessible transport of analytes or probes to the VMSF/electrode interface [35,36,37]. In addition, VMSFs have the electrostatic accumulation capacity for the target analyte due to the silanol groups on the walls and tiny nanochannels; at the same time, the insulating silica membrane can impede the ingress of interfering substances to the underlying electrode surface via charge, size, and lipophilicity interactions, showing good sensitivity, reproducibility, and long-term stability in real media analysis [38,39,40]. To enhance analytical sensitivity, electrocatalytic and/or conductive nanomaterials, such as metal nanoparticles [41], graphene quantum dots [42], and graphene nanosheets [43,44,45,46], have been incorporated into the inner space or bottom of VMSFs. Metal nanoparticles (e.g., gold and platinum) have been synthesized within the tiny nanochannels of VMSFs for the construction of attractive electrochemical sensors. To the best of our knowledge, CuNPs confined in the silica nanochannels of VMSFs have not yet been reported.

In this study, we synthesized electrodeposited CuNPs using an amino-functionalized, vertically ordered mesoporous silica film (NH_2_-VMSF) as a hard template. Their electrochemical performance with regard to NO_3_^−^ quantification in environmental water samples has been examined. Many uniform, tiny NH_2_-VMSF nanochannels favor the localized, stable growth of CuNPs, avoiding the use of any protective agent. The resulting CuNPs/NH_2_-VMSF-modified indium tin oxide (ITO) electrode, referred to as CuNPs/NH_2_-VMSF/ITO, exhibits superior analytical performance with respect to NO_3_^−^ due to the electrocatalytic properties of the CuNPs and the electrostatic accumulation ability of NH_2_-VMSF. Moreover, the CuNPs/NH_2_-VMSF/ITO sensor we developed exhibits excellent reproducibility, regeneration, and anti-interference capabilities, which have been successfully employed to accurately measure the concentration of NO_3_^−^ in diverse environmental water samples, including tap water, lake water, seawater, and rainwater.

## 2. Results and Discussion

### 2.1. Characterizations of NH_2_-VMSF/ITO and CuNPs/NH_2_-VMSF/ITO Electrodes

**Figure 1:**
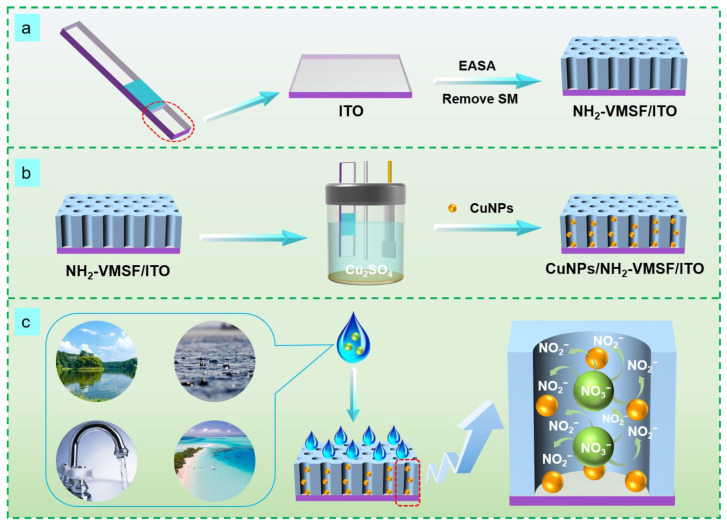
Schematic diagram of the preparation of an NH_2_-VMSF/ITO sensor (**a**) and a CuNPs/NH_2_-VMSF/ITO sensor (**b**) and the electrochemical reduction detection of NO_3_^−^ (**c**).

Figure 1 shows the schematic illustration of the preparation of a CuNPs/NH_2_-VMSF/ITO sensor and the electrochemical reduction detection of NO_3_^−^, which is divided into the following three sections: First, a binary film consisting of surfactant micelles (SM) and VMSF-bearing amino groups, abbreviated as SM@NH_2_-VMSF/ITO, was grown onto the patterned ITO electrode using the traditional electrochemical-assisted self-assembly (EASA) method [47,48] (Figure 1a). EASA combines electrochemical deposition and self-assembly techniques, inducing the self-assembly of SMs on the ITO electrode surface and the sol-gel process of the silane precursors within several seconds. SMs consisting of CTAB micelles are physically confined within the ultrasmall nanochannel space of the NH_2_-VMSF and can be excluded by simple solvent extraction [49]. The addition of (3-aminopropyl) triethoxysilane into the precursor solution can result in the silica nanochannels carrying amino groups [50]. The NH_2_-VMSF/ITO with an ultrasmall, open nanochannel array provided a confined nanospace for the stable synthesis of CuNPs with no protective agents. CuNPs were grown into the nanochannels of the NH_2_-VMSF using a controllable electrodeposition method to form the CuNPs/NH_2_-VMSF/ITO (Figure 1b), and their fabrication conditions (electrodeposition time) were also optimized. Not only could the CuNPs/NH_2_-VMSF/ITO enrich NO_3_^−^ through electrostatic interaction between the amino groups of the NH_2_-VMSF and NO_3_^−^, but it also exhibited the capacity to electrocatalytically reduce NO_3_^−^ via CuNPs. NO_3_^−^ could enter into the nanochannels of the NH_2_-VMSF and be electrochemically reduced to NO_2_^−^ in an acidic environment (Figure 1c), ultimately giving rise to the reductive peak and enabling the quantitative determination of NO_3_^−^. The anti-fouling properties of NH_2_-VMSF make the proposed CuNPs/NH_2_-VMSF/ITO sensor suitable for direct analysis of NO_3_^−^ in practical water samples.

**Figure 2:**
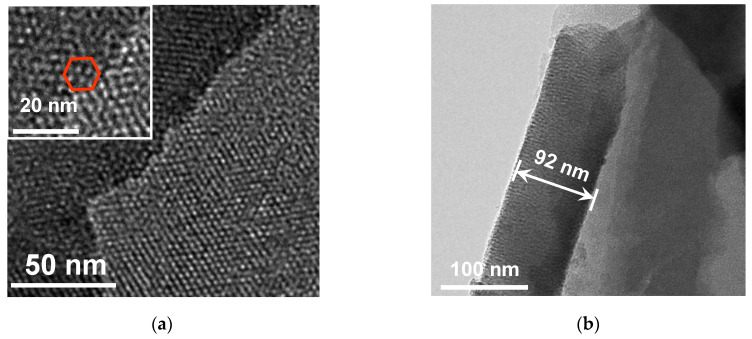

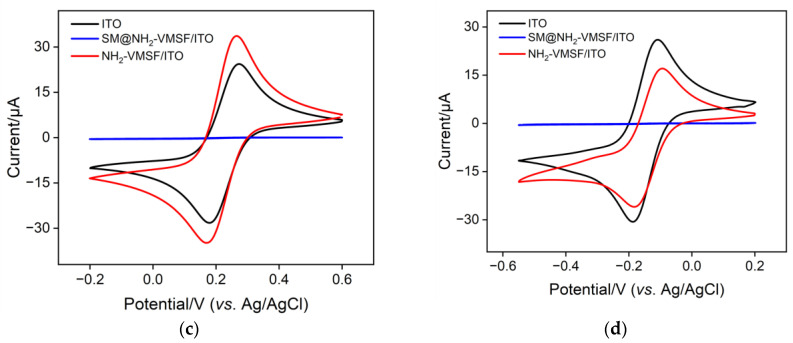
(**a**) Top-view and (**b**) cross-sectional TEM images of the NH_2_-VMSF. The inset in (**a**) is the corresponding magnified image showing hexagonally distributed nanopores. CV responses of ITO (black), VMSF/ITO (red) and SM@VMSF/ITO (blue) to 50 μM Fe(CN)_6_^3−^ (**c**) and 50 μM Ru(NH_3_)_6_^3+^ (**d**) at a scan rate of 50 mV/s. The supporting electrolytes for Fe(CN)_6_^3−^ and Ru(NH_3_)_6_^3+^ are 0.05 M KHP.

An NH_2_-VMSF layer grown on the ITO surface was investigated by transmission electron microscopy (TEM). As shown in Figure 2a, the NH_2_-VMSF, which was prepared using the electrochemically assisted self-assembly method with a high level of ordering, has a great deal of uniformly and hexagonally distributed nanopores (top-view TEM). The average pore diameter of the NH_2_-VMSF was in the range of 2~3 nm. Nanochannels oriented orthogonally to the NH_2_-VMSF surface were parallel to each other with a length of 92 nm (cross-sectional view SEM, Figure 2b). Modification of the NH_2_-VMSF layer with the permselective properties of the ITO was able to give rise to distinct electrochemical responses towards charged probes compared to a bare ITO electrode. Figure 2c,d shows the cyclic voltammetry (CV) curves of bare ITO, NH_2_-VMSF/ITO and SM@NH_2_-VMSF/ITO electrodes in a buffer solution containing either 50 μM Fe(CN)_6_^3−^ or 50 μM Ru(NH_3_)_6_^3+^. As can be seen, both Fe(CN)_6_^3−^ and Ru(NH_3_)_6_^3+^ were able to generate a pair of reversible redox peaks on the bare ITO (black curve). When insulating the NH_2_-VMSF with a SM inside the nanochannels, no Faradic current was measured at the SM@NH_2_-VMSF/ITO electrode (blue curve), because the templated SM molecules blocked access of charged hydrophilic probes; this further indicates that intact the NH_2_-VMSF homogeneously covers the whole ITO electrode surface. Effective exclusion of SMs from the nanochannels could be achieved using a HCl–ethanol solution to obtain the NH_2_-VMSF/ITO with recovered electrode accessibility [51]. Amino groups on the NH_2_-VMSF were exposed to the buffer solution and carried positive charges under the measured experimental conditions (pH = 7.0), leading to enhanced voltammetric currents for Fe(CN)_6_^3−^ (anodic peak current, *I*_ox_, 33.7 μA vs. 24.4 μA (bare ITO)), while decreasing signals for Ru(NH_3_)_6_^3+^ (*I*_ox_, 33.7 μA vs. 24.4 μA (bare ITO)) at the NH_2_-VMSF/ITO electrode. The values of the peak-to-peak separation obtained at the NH_2_-VMSF/ITO electrode were slightly larger than those at the bare ITO, suggesting the transport of probes into the nanochannels of NH_2_-VMSF is effective. This anion-selective permeability of the NH_2_-VMSF/ITO is due to the protonation of amino groups on the channel walls of the NH_2_-VMSF and to pronounced electrostatic interaction within the tiny space [52].

**Figure 3:**
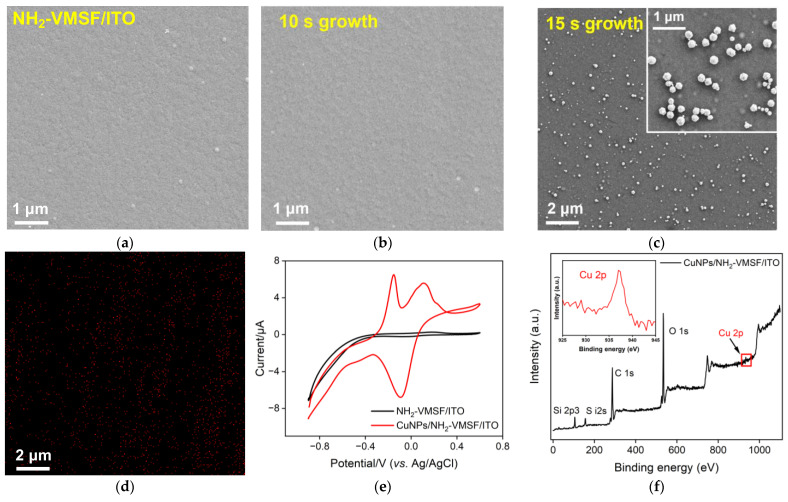
Top-view SEM images of the NH_2_-VMSF/ITO before (**a**) and after the confined growth of CuNPs for an electrodeposition time of 10 s (**b**) and 15 s (**c**). (**d**) The Cu element mapping image of the CuNPs/NH_2_-VMSF/ITO from Figure 2c. (**e**) CV curves of the NH_2_-VMSF/ITO and CuNPs/NH_2_-VMSF/ITO electrodes in a 0.1 M KCl solution. (**f**) XPS spectra of the CuNPs/NH_2_-VMSF/ITO; the inset is a magnified view of the red box in the figure.

The surface appearance of the NH_2_-VMSF before and after the confined growth of CuNPs for an electrodeposition time of 10 s and 15 s (abbreviated as CuNPs_10s_/NH_2_-VMSF/ITO and CuNPs_15s_/NH_2_-VMSF/ITO) was examined via scanning electron microscopy (SEM). As seen in Figure 3a–c, the CuNPs_10s_/NH_2_-VMSF/ITO gave rise to a smooth surface, which was similar to that of the NH_2_-VMSF/ITO, suggesting that electrodeposited CuNPs were inside the tiny nanochannels of the NH_2_-VMSF. However, numerous nanoparticles were observed at the surface of the CuNPs_15s_/NH_2_-VMSF/ITO (Figure 3c), which resulted from the formation of inhomogeneous, large CuNPs on the top surface of the NH_2_-VMSF when the electrodeposition time for the CuNPs was extended to 15 s (Figure 3d). Therefore, longer electrodeposition time can lead to the extended growth of CuNPs from the nanochannel to the surface of the NH_2_-VMSF/ITO. CuNPs on the surface of the NH_2_-VMSF/ITO are unstable and easily fall off the electrode surface, while those inside the silica nanochannels exhibit high stability due to the confinement effect. Considering the stability issue of the fabricated electrode, 10s was selected as the optimal electrodeposition time to guarantee the growth of CuNPs inside the nanochannels. Figure 3e shows CV curves for the NH_2_-VMSF/ITO and CuNPs/NH_2_-VMSF/ITO electrodes in a 0.1 M KCl solution. By comparison, the CuNPs/NH_2_-VMSF/ITO electrode exhibited the characteristic peaks of CuNPs, namely anodic peaks at −0.15 V and 0.10 V, corresponding to the oxidation of Cu(0) to Cu(I) and Cu(I) to Cu(II), and cathodic peaks at −0.09 V and −0.50 V, corresponding to the reduction of Cu(II) to Cu(I) and Cu(I) to Cu(0). Figure 3f shows the XPS spectrum of the CuNPs/NH_2_-VMSF/ITO, and the inset shows a magnified view of the peak of Cu 2p, demonstrating the presence of CuNPs. All of these results confirm the successful confinement of CuNPs inside the nanochannels of the NH_2_-VMSF via the electrodeposition procedure.

### 2.2. Electrocatalytic Reduction of NO_3_^−^ Using CuNPs/NH_2_-VMSF/ITO

**Figure 4:**
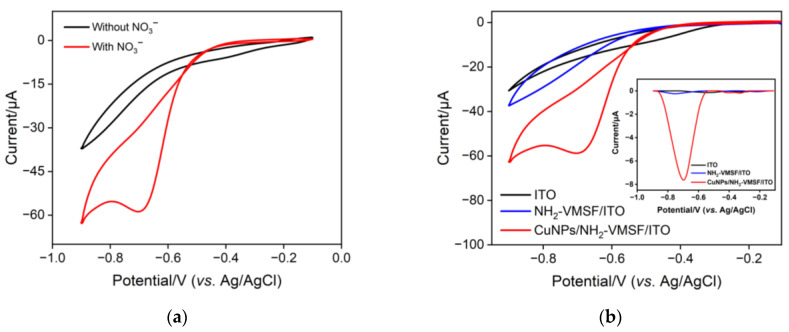
(**a**) CV curves of a fabricated CuNPs/NH_2_-VMSF/ITO electrode in 0.1 M Na_2_SO_4_ (pH = 3.0) in the absence and presence of 300 μM NO_3_^−^. (**b**) CV responses of bare ITO, NH_2_-VMSF/ITO, and CuNPs/NH_2_-VMSF/ITO electrodes towards 300 μM NO_3_^−^ in 0.1 M Na_2_SO_4_ (pH = 3.0). The inset in (**b**) shows the corresponding DPV curves for 300 μM NO_3_^−^.

Figure 4a depicts the electrochemical reduction ability of the fabricated CuNPs/NH_2_-VMSF/ITO electrode towards NO_3_^−^. Upon the addition of 300 μM NO_3_^−^ into a 0.1 M Na_2_SO_4_ (pH = 3.0) solution, an obvious cathodic peak was observed at the CuNPs/NH_2_-VMSF/ITO electrode, which was attributed to the electrocatalytic reduction of NO_3_^−^ at the fabricated electrode. Figure 4b compares the CV and DPV responses of the CuNPs/NH_2_-VMSF/ITO, NH_2_-VMSF/ITO and bare ITO electrodes towards 300 μM NO_3_^−^ in a 0.1 M Na_2_SO_4_ (pH = 3.0) solution. As shown, no cathodic peak signal was observed at the bare ITO electrode and a weak signal was obtained at the NH_2_-VMSF/ITO electrode due to the electrostatic interaction between the positively charged channel walls and the negatively charged NO_3_^−^. After electrodeposition of CuNPs into the nanochannels, the CuNPs/NH_2_-VMSF/ITO exhibited a significantly increased cathodic peak current for NO_3_^−^, which was attributed to the excellent electrocatalytic ability of CuNPs and the porous nanostructure of the NH_2_-VMSF for the growth of numerous CuNPs. Therefore, the inherent nanocatalytic properties of CuNPs and the electrostatic accumulation ability were combined to obtain a highly sensitive determination of NO_3_^−^, showing superior analytical performance.

**Figure 5:**
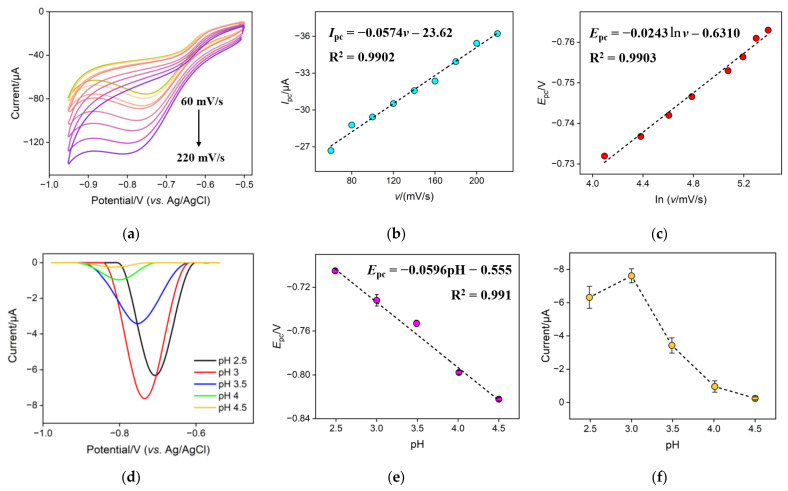
(**a**) CV curves for 300 μM NO_3_^−^ in 0.1 M Na_2_SO_4_ (pH = 3.0) at the CuNPs/NH_2_-VMSF/ITO under different scan rates (from top to bottom: 60, 80, 100, 120, 140, 160, 180, 200, and 220 mV/s). (**b**) The plot of the cathodic peak current (*I*_pc_) obtained from (**a**) against scan rate (*v*). (**c**) The plot of the cathodic peak (*E*_pc_) obtained from (**a**) against the natural logarithm of the scan rate (ln*v*). (**d**) DPV responses of the CuNPs/NH_2_-VMSF/ITO for 300 μM NO_3_^−^ in 0.1 M Na_2_SO_4_, adjusted to various pH values. The plots of the *E*_pc_ (**e**) and cathodic peak current (**f**) obtained from (**d**) against the pH value.

The mechanism for the electrocatalytic reduction of NO_3_^−^ on the CuNPs/NH_2_-VMSF/ITO electrode was investigated via CV and differential pulse voltammetry (DPV). Figure 5a shows the CV curves of 300 μM NO_3_^−^ in 0.1 M Na_2_SO_4_ (pH = 3.0) at the CuNPs/NH_2_-VMSF/ITO under different scan rates. The cathodic peak current (*I*_pc_) and cathodic peak (*E*_pc_) of 300 μM NO_3_^−^ at the CuNPs/NH_2_-VMSF/ITO were extracted from Figure 5a and plotted as a function of scan rate (*v*) and natural logarithm (ln *v*), respectively. As shown in Figure 5b, the *I*_pc_ was linearly proportional to the *v* within the range of 60 to 220 mV/s, suggesting a surface-controlled electrocatalytic reduction process of NO_3_^−^ at the CuNPs/NH_2_-VMSF/ITO. The relation between *E*_pc_ and ln *v* was linear as shown in Figure 5c and can be expressed as the following equation:

(1) $$E_{pc}=-0.0243\ln v-0.6310\;\left(R^{2}=0.9903\right)$$

The Laviron equation was used as follows to describe the relationship between *E*_pc_ and ln *v*:

(2) $$E_{pc}=E_{0}^{\prime }+\left(\frac{\mathrm{RT}}{\alpha nF}\right)\ln\left(\frac{RTK_{s}}{\alpha nF}\right)-\left(\frac{RT}{\alpha nF}\right)\ln v$$

where $E_{0}^{\prime }$, *K*s, *α*, and *n* are the standard electrode potential, the standard heterogeneous rate constant, the transfer coefficient, and the number of electrons involved in the rate-determining step, respectively. Other symbols have their usual physical meanings: *R*, gas constant (8.314 J moL^−1^ K^−1^); *T*, absolute temperature (298 K); *F*, Faraday constant (96,485 C moL^−1^).

The following can be deduced from Equations (1) and (2):

(3) $$\left(\frac{RT}{\alpha nF}\right)=0.0243\;$$

According to Equation (3), the value of *αn* was calculated to be 1.05. Given a α of 0.5 in the completely irreversible electrochemical reaction, *n* was calculated to be 2.1, indicating that the reduction process of NO_3_^−^ involves two electrons.

The slope of the fitting linear relationship between *E*_pc_ and pH could be used to determine the ratio of electrons and protons participating in the electrochemical reaction on the electrode surface. Figure 5d shows the DPV curves of the CuNPs/NH_2_-VMSF/ITO electrode for 300 μM NO_3_^−^ in 0.1 M Na_2_SO_4_, adjusted to various pH values. As the curves demonstrate, *E*_pc_ became more negative as the pH increased. The good linear relationship shown in Figure 5e in the range of 2.5 to 4.5 can be expressed as follows:

(4) $$E_{pc}=-0.0596pH-0.555\left(R^{2}=0.991\right)$$

Given that $\frac{dE_{pc}}{dpH}=2.303\frac{mRT}{nF}$, where *m* is the number of protons and the other symbols are the same as above, the calculated m/n for the NO_3_^−^ reduction process was 1.01, indicating equal involvement of protons and electrons in the electrochemical reduction of NO_3_^−^. Combining an n of 2 as obtained above, it could be inferred that the electrochemical reduction reaction of NO_3_^−^ is a two-electron coupled, two-proton process, which can be shown as follows:

(5) $${NO}_{3}^{-}+2H^{+}+2e^{-}\to {NO}_{2}^{-}+H_{2}O$$

### 2.3. Influence of Experimental Conditions on Electrochemical Detection of NO_3_^−^

To obtain good analytical performance, the influence of the pH value of the Na_2_SO_4_ solution on the reduction of NO_3_^−^ was investigated. Figure 5f shows the cathodic peak current for 300 μM NO_3_^−^ in 0.1 M Na_2_SO_4_ at the CuNPs/NH_2_-VMSF/ITO at different pH values. The cathodic peak current initially increased with increasing pH, reaching a maximum at pH 3, and then decreased as the pH continued to increase. When the pH was less than 3, a hydrogen evolution reaction occurred at a less negative potential in strongly acidic media, which could affect the reduction of NO_3_^−^. When the pH was greater than 3, a decrease in the cathodic peak current of NO_3_^−^ was found. This is because the hydrogen ions participate in the electrochemical reduction reaction of NO_3_^−^ to NO_2_^−^. An increase in pH made the chemical equilibrium (Equation (5)) shift to the left, leading to the decreased cathodic peak current. Therefore, pH 3 was selected as the optimum condition.

**Figure 6:**
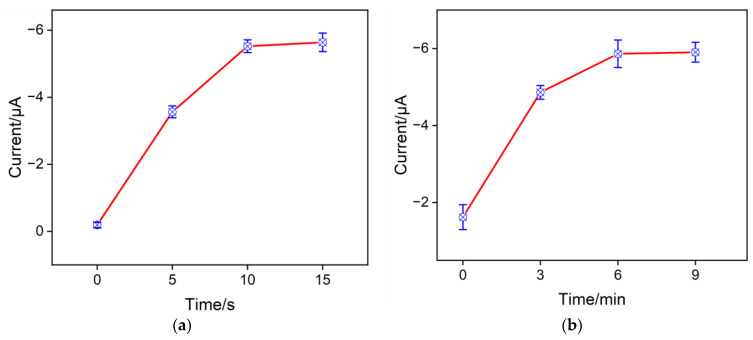
Effects of electrodeposition time (**a**) on the growth of CuNPs and of mechanical stirring time (**b**) for the preconcentration of NO_3_^−^ on the electrochemical responses for 100 μM NO_3_^−^ at the CuNPs/NH_2_-VMSF/ITO in a Na_2_SO_4_ (0.1 M, pH = 3) solution.

Optimizing the amount of CuNPs inside the nanochannels of the NH_2_-VMSF was crucial to the effective accumulation of NO_3_^−^ for achieving the highest sensitivity. The electrodeposition time of CuNPs can be used for determining the amount of CuNPs inside the nanochannels, which was studied in Figure 6a. As shown here, when the electrodeposition time of the CuNPs increased from 0 s to 10 s, the electrochemical reduction signal of NO_3_^−^ gradually improved, because having more CuNPs inside the nanochannels enhanced the electrocatalytic capacity for NO_3_^−^ reduction. But further increasing the electrodeposition time causes excessive aggregation of CuNPs on the outer surface of the NH_2_-VMSF channels. Therefore, the optimal electrodeposition time for the growth of CuNPs was achieved at 10 s. The inner walls of the NH_2_-VMSF channels are rich in amino groups, which carry positive charges in acidic environments and exhibit electrostatic adsorption towards NO_3_^−^. When the CuNPs/NH_2_-VMSF/ITO was magnetically stirred in a buffer solution containing 100 μM NO_3_^−^, the achieved cathodic peak current signal increased significantly from 0 to 6 min (Figure 6b), which was due to more NO_3_^−^ having diffused to the underlying electrode surface through the nanochannels of the NH_2_-VMSF. After 6 min, the cathodic peak current of NO_3_^−^ remained unchanged. Therefore, the accumulation time of 6 min was employed for the subsequent test.

### 2.4. Electroanalytical Performance of NO_3_^−^ Using CuNPs/NH_2_-VMSF/ITO

**Figure 7:**
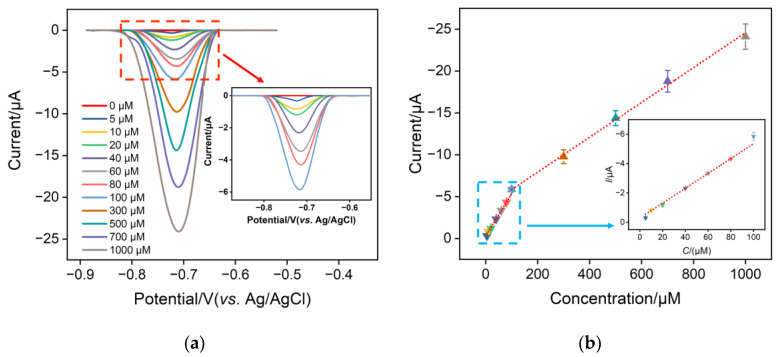
(**a**) DPV responses of the CuNPs/NH_2_-VMSF/ITO to the successive addition of various concentrations of NO_3_^−^ in a Na_2_SO_4_ (0.1 M, pH 3.0) solution. The concentrations of NO_3_^−^ range from 5 μM to 1000 μM. (**b**) The cathodic peak current—concentration plot for the CuNPs/NH_2_-VMSF/ITO electrode with the addition of various concentrations of NO_3_^−^ in a Na_2_SO_4_ (0.1 M, pH 3.0) solution. Insets in (**a**,**b**) represent the corresponding amplified curves in the low concentration range, and the error bars in (**b**) represent the standard deviations of three measurements.

The differential pulse voltammetry (DPV) technique was used to determine different concentrations of NO_3_^−^ in a 0.1 M Na_2_SO_4_ solution using the CuNPs/NH_2_-VMSF/ITO electrode. Figure 7 shows the DPV signals and calibration curves of NO_3_^−^ in the range of 5 μM^−1^ mM. As presented, the cathodic peak current of NO_3_^−^ tested at the CuNPs/NH_2_-VMSF/ITO electrode grew linearly with concentration. The linear regression equation in the range of 5–100 μM can be expressed as *I* (μA) = 0.051 *C* (μM) + 0.29 (*R*^2^ = 0.996), and the other linear regression equation in the range of 100–1000 μM as *I* (μA) = 0.021 *C* (μM) + 3.77 (*R*^2^ = 0.998). The limit of detection (LOD) was estimated to be 2.3 μM at a signal-to-noise ratio of three using the formula of LOD = 3SD/*k* (where SD and *k* are the standard deviation of the blank solution and the slope of the calibration curve, respectively). Note that the LOD value is far below the concentration limit (806 μM) for NO_3_^−^ in the water quality standard specified by World Health Organization. Moreover, the proposed CuNPs/NH_2_-VMSF/ITO has several advantages over other electrodes reported in the literature, such as a lower LOD, a wider dynamic linear range, and easy fabrication steps (Table 1).

### 2.5. Anti-Interference, Regeneration, Reproducibility, and Stability of CuNPs/NH_2_-VMSF/ITO

**Figure 8:**
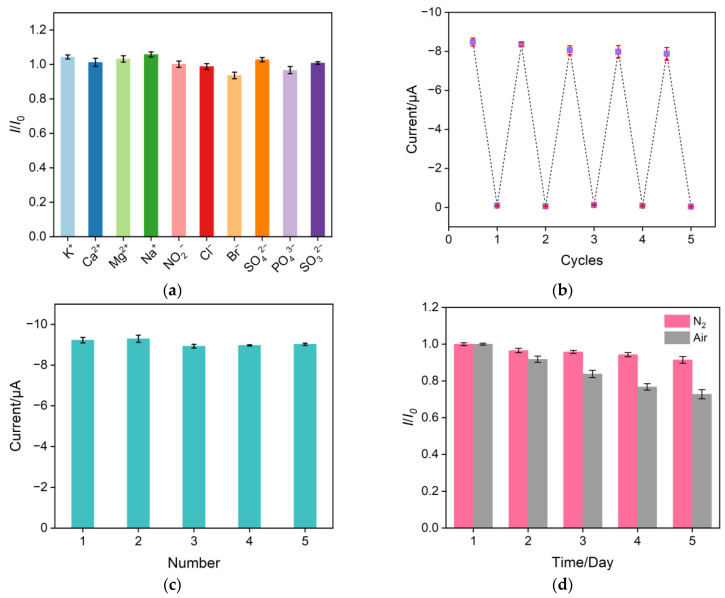
(**a**) Cathodic peak current ratio obtained at the developed CuNPs/NH_2_-VMSF/ITO electrode for the detection of 100 μM NO_3_^−^ before (*I*_0_) and after (*I*) the addition of 1 mM of various interfering substances to a Na_2_SO_4_ solution (0.1 M pH = 3). (**b**) DPV signals of 300 μM NO_3_^−^ measured repeatedly after multiple elutions of the CuNPs/NH_2_-VMSF/ITO electrode. (**c**) DPV signals of 300 μM NO_3_^−^ in a Na_2_SO_4_ solution (0.1 M pH = 3) measured using five different electrodes prepared in parallel. (**d**) DPV signals of the CuNPs/NH_2_-VMSF/ITO electrodes for the detection of 300 μM NO_3_^−^ in a 0.1 M Na_2_SO_4_ (pH = 3) solution after storage for different numbers of days in air and nitrogen atmospheres, respectively. The error bars represent the standard deviations of three measurements.

The selectivity of the prepared CuNPs/NH_2_-VMSF/ITO sensor for NO_3_^−^ detection was evaluated in the presence of common interfering ions, such as 1 mM Na^2+^, K^+^, Ca^2+^, Mg^2+^, Na^+^, NO_2_^−^, Cl^−^, Br^−^, SO_4_^2−^, PO_4_^3−^, and SO_3_^2−^. As shown in Figure 8a, a ten-fold concentration of these interfering ions produced minimal interference for the determination of 100 μM NO_3_^−^ at the CuNPs/NH_2_-VMSF/ITO, indicating the good selectivity and anti-interference ability of the proposed sensor. To assess the electrode’s regeneration capacity, the same CuNPs/NH_2_-VMSF/ITO electrode was used to repeatedly measure 300 μM NO_3_^−^; the used electrode was washed with a 0.1 M HCl–ethanol solution for 5 min prior to testing. As shown in Figure 8b, no significant decrease in the current signal was observed at our fabricated sensor after five-time elution, demonstrating the excellent regeneration ability of the sensor. Five batches of CuNPs/NH_2_-VMSF/ITO electrodes were prepared under the same conditions and used to test 300 μM NO_3_^−^ in order to examine the reproducibility of the electrode. The calculated relative standard deviation (RSD) of the measured results from the five electrodes was 1.8% (Figure 8c), confirming the high reproducibility of the CuNPs/NH_2_-VMSF/ITO electrode. Additionally, the stability of the fabricated CuNPs/NH_2_-VMSF/ITO electrode was studied by comparing the initial cathodic peak current of NO_3_^−^ with that obtained after five days in storage. The data shown in Figure 8d prove the good stability of CuNPs/NH_2_-VMSF/ITO electrode under a nitrogen atmosphere compared with storage under an air atmosphere.

### 2.6. Direct Analysis of NO_3_^−^ in Water Samples 

According to the previous reports, NH_2_-VMSF serves as a good anti-fouling protective layer on the electrode surface and has been used to design many electrochemical sensors in rather complicated real samples [38]. Environmental water samples including tap water, rainwater, lake water, and seawater were selected to validate the practical applicability of our fabricated CuNPs/NH_2_-VMSF/ITO electrode. The pH of these collected water samples was adjusted to 3 using sulfuric acid, and several known concentrations of NO_3_^−^ were added. The CuNPs/NH_2_-VMSF/ITO electrode was then used to analyze the above samples using the DPV technique. Table 2 shows the quantitative results for NO_3_^−^ in water samples using the standard addition method. As shown, our proposed CuNPs/NH_2_-VMSF/ITO electrode exhibited excellent recovery values ranging from 97.8% to 109%, demonstrating the good analytical performance of the developed sensor in real water samples.

## 3. Materials and Methods

### 3.1. Chemicals and Instrumentations

All analytical grade chemicals and reagents in this study were used as received without further purification. Ultrapure water was obtained from the Millipore Milli-Q system (18 MΩ cm). Tetraethoxysilane (TEOS), cetyltrimethylammonium bromide (CTAB), and (3-aminopropyl) triethoxysilane (APTES) were purchased from Sigma-Aldrich. Potassium ferricyanide (K_3_[Fe(CN)_6_]), hexaammineruthenium (III) chloride ([Ru(NH_3_)_6_Cl_3_]), and acetone were ordered from Shanghai Aladdin Biochemical Technology Co., Ltd. Sodium sulfate (Na_2_SO_4_), sodium sulfite (Na_2_SO_3_), sodium nitrite (NaNO_2_), sodium phosphate (Na_3_PO_4_), sodium chloride (NaCl), potassium chloride (KCl), potassium bromide (KBr), potassium nitrate (KNO_3_), and copper (II) sulfate pentahydrate (CuSO_4_·5H_2_O) were bought from Shanghai Macklin Biochemical Technology Co., Ltd. (Shanghai, China) Indium tin oxide (ITO) conductive glass (surface resistivity < 17 Ω/square, thickness of 100 ± 20 nm) were purchased from Zhuhai Kaivo Optoelectronics Technology Co., Ltd. (Zhuhai, China) The ITO glass was sonicated with 1 M aqueous sodium hydroxide for two hours, followed by acetone, ethanol, and deionized water sonication for ten minutes each. Finally, the ITO glass was dried at 60 °C prior to use.

The shape and thickness of the NH_2_-VMSF were determined using transmission electron microscopy (TEM, HT7700, Hitachi, Japan) and scanning electron microscopy (SEM, SU8010, Hitachi, Japan) at accelerating voltages of 200 kV and 5 kV, respectively. A PHI5300 electron spectrometer (PE Ltd., Boston, MA, USA) was used to conduct an X-ray photoelectron spectroscopy (XPS) examination with 250 W, 14 kV Mg K radiation. Electrochemical impedance spectroscopy (EIS), cyclic voltammetry (CV), and differential pulse voltammetry (DPV) measurements were carried out using an Autolab electrochemical workstation (PGSTAT302N, Metrohm, Switzerland). Measurements of electrochemical reactions were performed using a conventional three-electrode system. A bare ITO or modified ITO (0.5 × 1 cm^2^), an Ag/AgCl (saturated with KCl solution), and a platinum wire were selected as the working electrode, the reference electrode, and the counter electrode, respectively.

### 3.2. Preparation of the NH_2_-VMSF/ITO Electrode

The NH_2_-VMSF was grown on a conductive ITO electrode using the electrochemically assisted self-assembly (EASA) method [35]. A mixed solution composed of 20 mL ethanol, 20 mL 0.1 M NaNO_3_, 13.6 mM “TEOS + APTES” (9:1 molar ratio), and 4.35 mM CTAB were first prepared. After adjusting the pH to 3.0 with hydrochloric acid (HCl, 3 M), the precursor solution was stirred for 2.5 h at room temperature. A clean ITO (0.5 × 1 cm^2^) was immersed into the precursor solution and electrodeposited at a constant current of −0.35 mA for 10 s. The resulting electrode was immediately removed from the growth solution, thoroughly rinsed with ultrapure water, dried under a nitrogen atmosphere, and aged overnight at 120 °C. During the preparation process, a surfactant micelle (SM) consisting of CTAB served as a template and remained in the nanochannels of the NH_2_-VMSF; this was given the designation SM@NH_2_-VMSF/ITO. SMs can be easily eliminated by stirring in 50 mL of an ethanol solution containing 0.1 M hydrochloric acid for 5 min. The resulting electrode was designated as the NH_2_-VMSF/ITO electrode.

### 3.3. Electrochemical Deposition of CuNPs

The electrochemical deposition of CuNPs into the NH_2_-VMSF was modified slightly according to reference [56] as follows: 0.5 mmol CuSO_4_·5H_2_O was added to 50 mL of a 0.1 M sulfuric acid solution and sonicated for 5 min to obtain the electrodeposition solution. Then, the NH_2_-VMSF/ITO/ITO electrode was placed in the above electrodeposition solution and was subjected to a constant potential of −0.6 V for 10 s using a platinum sheet as the counter electrode and Ag/AgCl as the reference electrode. Finally, the resulting electrode was rinsed with ultrapure water and blow-dried with nitrogen gas, yielding the CuNPs/NH_2_-VMSF/ITO electrode.

### 3.4. Detection of NO_3_^−^

Before the electrochemical test, the Na_2_SO_4_ solution (0.1 M and pH adjusted to 3.0 with sulfuric acid) was deoxygenated by bubbling nitrogen gas in the solution for 30 min. Various concentrations of NO_3_^−^ were added to the above solution and determined using the CuNPs/NH_2_-VMSF/ITO electrode under a nitrogen atmosphere. The DPV parameters included a step potential of 0.005 V, a pulse amplitude of 0.05 V, an interpulse time of 0.2 s, and a pulse time of 0.05 s.

### 3.5. Actual Sample Testing

Tap water, rainwater, lake water, and seawater were selected as actual samples to verify the accuracy of the CuNPs/NH_2_-VMSF/ITO sensor using the standard addition method. Tap water, lake water, and rainwater in the samples were sourced locally (Hangzhou, China), and seawater produced in Qingdao (Shandong, China) was purchased from Taobao. The pH of these environmental water samples was adjusted to 3 using sulfuric acid (0.1 M) without dilution. Different concentrations of NO_3_^−^ were added to the water samples and then determined using the CuNPs/NH_2_-VMSF/ITO sensor.

## 4. Conclusions

A simple and highly sensitive NO_3_^−^ electrochemical sensor was developed based on an NH_2_-VMSF and CuNPs confined in the nanochannels. Physical confinement of CuNPs was achieved via a controllable one-step electrodeposition procedure. The immobilization of the CuNPs in the tiny nanochannels endows the electrode with the electrocatalytic capacity for reducing NO_3_^−^ and conducting highly sensitive NO_3_^−^ measurements. Not only can the NH_2_-VMSF serve as a hard template for the stable growth of CuNPs, but it can also provide the electrostatic accumulation capacity for the target NO_3_^−^. The detection limit for this kind of CuNPs/NH_2_-VMSF/ITO sensor is as low as 2.3 μM with a linear range extending from 5.0 μM to 1 mM for NO_3_^−^ determinations. Furthermore, direct analysis of NO_3_^−^ concentrations in various environmental samples using our proposed sensor was evaluated, revealing acceptable accuracy and great promise for fast NO_3_^−^ monitoring in samples of polluted water such as sewage.

## Figures and Tables

**Table 1:** Analytical results of several modified electrodes for the detection of NO_3_^−^.

Electrode	Detection Method	Linear Range (μM)	LOD (μM)	Real Sample	Ref.
Cu-NWs/copper tape	LSV	10.0–1.5 × 10^3^	9.1	river, rainwater and drinking	[53]
PEG-SH/SePs/AuNPs/PCE	DPV	16.0–5 × 10^3^	8.6	lake water	[54]
Cu@TiO_2_-Nf/PAR/GCE	DPV	5.0–7.5 × 10^3^	2.1	river water and tap water	[55]
Cu/MWCNT/RGO/GCE	SWV	0.1 × 75	0.02	mineral water tap water and	[56]
Pt/Ag/ITO	CV	266–4.4 × 10^3^	134.0	simulated ground water	[57]
IIP-Cu-NPs/PANI/GCE	EIS LSV	1.0–1 × 10^3^	31.0 5.0	mineral water well water	[58]
Cu-NWs/Cu wire	LSV	50.0–600	12.2	not shown	[59]
CuNPs/NH_2_-VMSF/ITO	DPV	5.0–1 × 10^3^	2.3	tap water, pond water, seawater and rainwater	This work

Cu-NWs: copper nanowires. PEG-SH: poly(ethyleneglycol) methylether thiol. SePs: selenium particles. AuNPs: gold nanoparticles. PCE: paper carbon electrode. Nf: nafion. PAR: polyalizarin yellowe R. MWCNT: multiwall carbon nanotubes. RGO: reduced graphene oxide. IIP: ion-imprinted polymer. PANI: polyaniline.

**Table 2:** Quantification of NO_3_^−^ in real samples using CuNPs/NH_2_-VMSF/ITO electrode.

Sample	Added (μM)	Found (μM)	Recovery (%)	RSD (%, *n* = 3)
	10.0	10.2	102	0.9
Tap Water	100	103	103	1.9
	500	495	99.0	3.1
	30.0	32.8	109	3.9
Pond Water	100	97.8	97.8	3.4
	500	503	101	1.2
	30.0	29.9	99.6	2.6
Rainwater	100	100	100	1.6
	500	505	101	0.8
	30.0	30.9	103	2.3
Seawater	100	101	101	3.4
	500	518	104	1.3

## Data Availability

The data presented in this study are available on request from the corresponding author.
